# The Inside Mystery of Jejunal Gastrointestinal Stromal Tumor: A Rare Case Report and Review of the Literature

**DOI:** 10.1155/2011/985242

**Published:** 2011-07-02

**Authors:** A. K. Dhull, V. Kaushal, R. Dhankhar, R. Atri, H. Singh, N. Marwah

**Affiliations:** ^1^Department of Radiation Oncology, PGIMS, P.O. Box 100, Rohtak 124001, India; ^2^Department of Pathology, PGIMS, Rohtak 124001, India

## Abstract

Gastrointestinal stromal tumors (GISTs) are malignant and rare form of soft tissue sarcoma of the digestive tract. The incidence of gastrointestinal stromal tumors is very low Kramer et al. 2005 Jejunal GISTs are extremely rare. Here we present a rare case of jejunal GIST with unusually large size at presentation. The patient presented with severe abdomen pain, exophytic growth, and dimorphic anemia. Surgical resection of the tumor was carried out, and operative findings revealed a 15 × 10 cm growth, arising from serosal surface of jejunum, at the antimesenteric surface. Diagnosis in this case was made by subjecting the resected specimen to immunohistochemical analysis. In view of large size of the resected tumor, and high-risk histopathological features, imatinib mesylate 400 mg once daily was given as adjuvant chemotherapy. Patient is asymptomatic without any evidence of tumor recurrence after six months of postoperative followup. Imatinib as such is recommended in metastatic, residual or recurrent cases of GISTs or which are surgically not removable; however, recent recommendations suggests the use of imatinib mesylate after radical surgery in high-risk cases, because it has shown a significant decrease in the recurrence rate, and the Food and Drug Administration (FDA) has also approved the use of imatinib as adjuvant therapy after complete resection of localized, primary GIST.

## 1. Introduction

Gastrointestinal stromal tumors (GISTs) are malignant and rare form of soft tissue sarcoma of the digestive tract. Most common site of presentation is stomach, but it can crop up anywhere in the digestive tract. Two-thirds of GISTs occur in the stomach [1] while about one-fourth develop in the small intestine, usually in the duodenum [2]. GISTs are uncommon mesenchymal neoplasms of the alimentary tract. The incidence of GIST is very low (i.e., 2 in 1,00,000) while jejunal GIST is extremely rare accounting for 0.1–3% of all gastrointestinal (GI) tumors [3]. Usually they are asymptomatic but can present as abdominal pain, bleeding, or mechanical obstruction. Exophytic growths of these tumors have been noted in 18–30% of cases [4]. We present a rare case of jejunal GIST with unusual size of presentation and with history of severe pain abdomen, exophytic growth, and dimorphic anemia.

## 2. Case Summary

A 38-year-male patient presented with two-month-old history of pain abdomen and feeling of heaviness in right lower abdomen. Pain was severe in intensity and relieved only after taking medication. Patient also gave a history of lump in the abdomen. General physical and systemic examination was normal. Local examination of abdomen and pelvis revealed a hard mass measuring around 15 × 10 cm in size in the right iliac region. Complete hemogram of the patient was suggestive of dimorphic iron deficiency anemia with thrombocytosis. Routine blood biochemistry parameters of the patient were within normal limits. 

**Figure 1:**
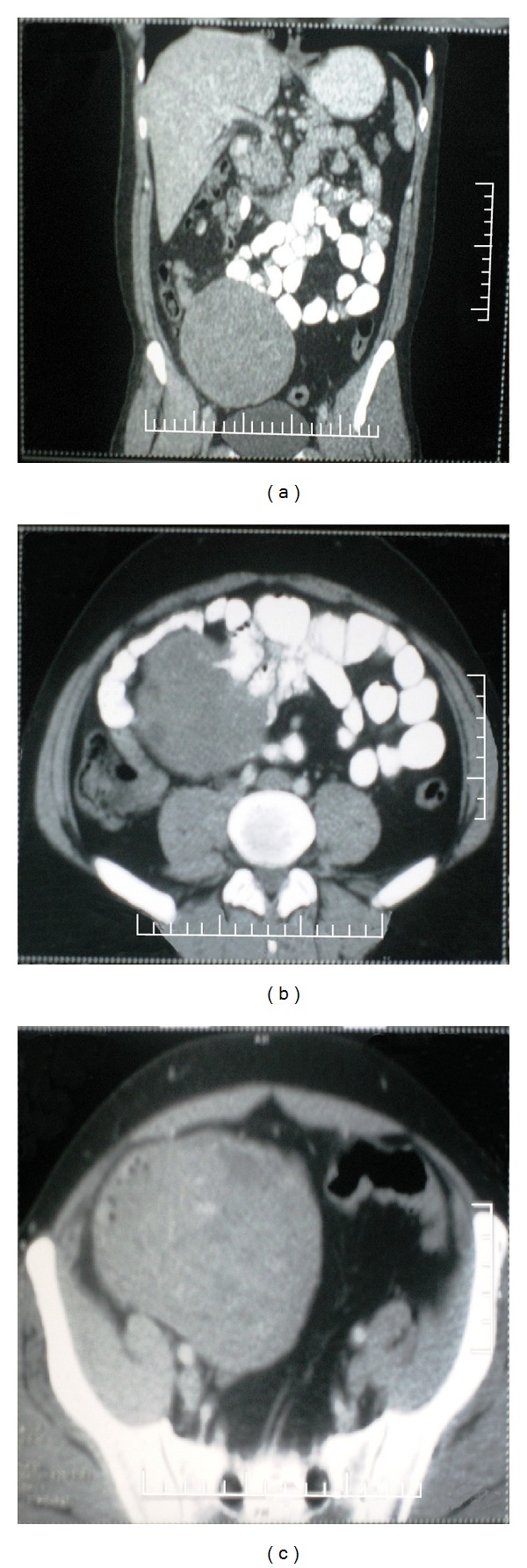
Computed tomography enteroclysis showing a large well-defined heterodense ovoid space occupying lesion measuring 15 × 10 cm with clumped bowel loops seen in right iliac fossa.

Abdominopelvic ultrasonography revealed a large hypoechoic lesion, in the right iliac region. No mucosal lesion was seen on upper and lower gastrointestinal endoscopy. Computed tomography enteroclysis showed a large well-defined heterodense ovoid space occupying lesion measuring 15 × 10 cm with clumped bowel loops seen in right iliac fossa with intraluminal protrusion and exophytic growth pattern, without lymphadenopathy or metastatic disease (Figures 1(a), 1(b) and 1(c)). Surgical resection of the tumor was carried out, and operative findings revealed a 15 × 10 cm growth, arising from serosal surface of jejunal junction, at the antimesenteric surface. 

**Figure 2:**
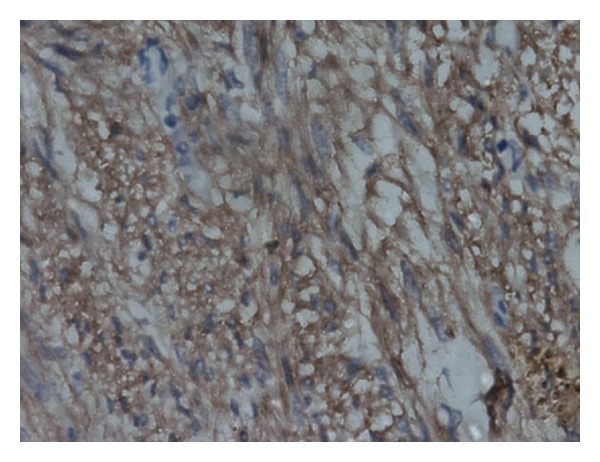
Photomicrograph (IHC stain; original magnification ×400) of immunohistochemical study showing tumor cells positive for KIT (CD117).

**Figure 3:**
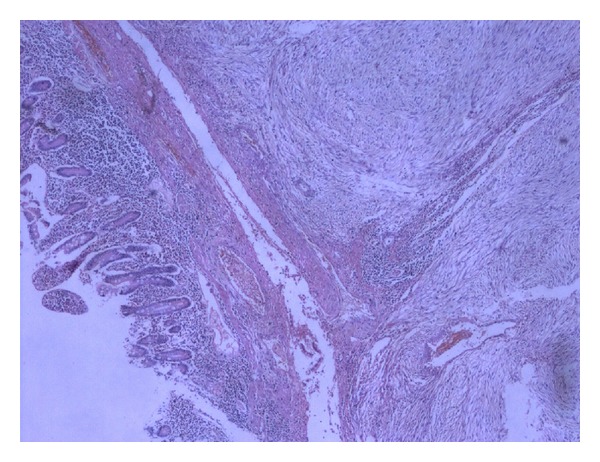
Photomicrograph (H&E stain; original magnification ×40) showing unremarkable overlying mucosa, submucosa, muscularis propria, and serosa showing infiltration by tumor.

**Figure 4:**
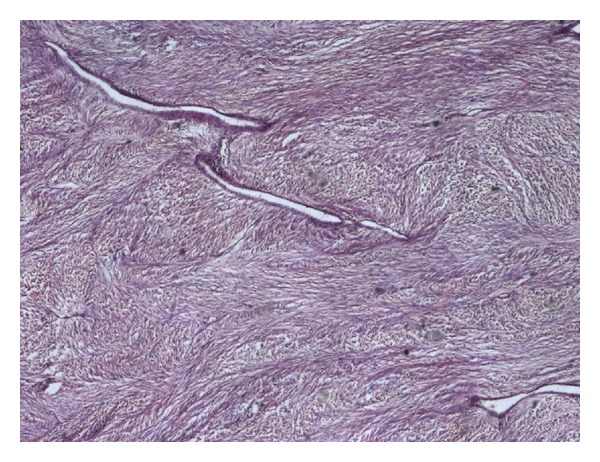
Photomicrograph (H&E stain; original magnification ×40) showing oval to spindle cells arranged in fascicles.

Macroscopically cut surface was greyish white with the areas of haemorrhage and cystic degeneration. The neoplastic cells illustrated wide spread positivity for immunocytochemical stain, CD117 (a proto-oncogene also known as KIT; Figure 2). Cells were found to be negative for CK and LCA immunochemical stains. Histopathologically, the overlying mucosa was unremarkable, underlying submucosa, muscularis propria, and serosa shows infiltration by plump spindle cells with bilateral surgical margins free from tumor infiltration (Figures 3 and 4). The histopathological appearance and immunohistochemical profile of the mass confirmed it to be a high-grade gastrointestinal stromal tumor (size being more than 10 cms). The patient had an uneventful postoperative course. In view of large size of the resected tumor, and high-risk histopathological features, imatinib mesylate 400 mg once daily was given as adjuvant chemotherapy, and patient is asymptomatic without any evidence of tumor recurrence after six months of postoperative followup.

## 3. Discussion

The incidence of GIST is very low, that is, 2 in 1,00,000 while jejunal GISTs are extremely rare, [3] accounting for 0.1–3% of all gastrointestinal (GI) tumors [5]. The most common site of presentation is stomach, but it can crop up anywhere in the digestive tract. Two-thirds of GISTs occur in the stomach [1] while about one-fourth develop in the small intestine, usually in duodenum [2]. As per the literature, jejunal GISTs are the rarest type among all types of GIST [3]. The most common clinical manifestation for symptomatic GISTs is occult gastrointestinal (GI) bleeding from mucosal ulceration and pain abdomen [6]. Five percent of GI hemorrhage is obscure in nature, and GISTs have been described as one of the cause [7]. 

Various unusual forms and variants of the GISTs have been described, including jejunal GIST. Immunocytochemical studies showed KIT (CD117) and CD34 immunopositivity, attributes that are restricted in the gut to the interstitial cells of Cajal [8]. The interstitial cells of Cajal are mesodermal derivatives that become associated with the autonomic myenteric plexus during development and are thought to regulate peristalsis [8]. Today, the majority of alimentary tract intramural tumors are interpreted as GISTs, including tumors formerly referred to as gastrointestinal autonomic nerve (GAN) tumor and plexosarcoma. Activating mutations of the KIT gene are often found in GISTs. GISTs are not a homogeneous group of neoplasms, however. Immunocytochemically, some show differentiation toward smooth muscle, others toward nerve, some toward histiocytes, a small group toward smooth muscle and nerve, and another small group shows no differentiation. GISTs also show strong site-dependent genetic heterogeneity [8]. The tumor is a major or minor component of certain rare syndromes, familial, and nonfamilial functioning paraganglioma, and GIST are uncommon tumors that occur mostly in a sporadic and isolated form, occasionally as components of multiple neoplasia syndromes, either separately or together. Separately, they occur in several inherited syndromes including multiple endocrine neoplasia [8], the GIST, lentigines, and mast cell tumor syndrome. Together, they are variably prominent components of three syndromes: the familial paraganglioma and gastric GIST syndrome, neurofibromatosis type 1, and the Carney triad (syndrome with paraganglioma-jejunal GIST combination). The two former conditions are inherited as autosomal dominant traits; the later does not appear to be inherited and affects young women predominantly [8]. 

The main differential diagnosis of benign or a small-sized malignant GIST is gastrointestinal schwannomas [9]. Gastric schwannomas are divided into two major subgroups as mesenchymal or neuroectodermal [9]. It is crucial to differentiate GIST from GI schwannomas, as GI schwannomas are biologically benign tumors with an excellent prognosis [10] whereas GISTs carry a relatively higher malignant potential [11]. 20–30% of the GISTs are malignant in nature but most (70–80%) are benign [5]. On CT imaging, GI schwannomas have homogenous attenuation and can be readily differentiated from a large benign or malignant GIST, which demonstrates heterogeneous enhancement due to haemorrhage, necrosis, and intralesional cystic changes [9]. Gastrointestinal schwannomas are distinctive from conventional schwannomas, which originate from CNS or soft tissues [9]. Diagnosis on CT imaging becomes very difficult if the mass is small and lacks ulceration and necrosis. In such cases histopathology is often required for confirming the diagnosis.

Surgery is the primary treatment of choice and for a long time has been the only effective treatment for GIST with overall 5-year survival rates of 45–55% until 2001 when Imatinib, a small molecule inhibiting the kinase activity of *c-kit*, *PDGFR*α**, and *BCR-ABL* was recognized to be highly effective in metastasized GIST and revolutionised the treatment of metastasized and/or unresectable GIST [12]. However, an uncertainty remains whether an individual tumor behaves benign or whether it has the potential for metastatic spread. To date, tumor size and the mitotic index shape the risk sheets applied in clinical practice [12]. In fact, local recurrence and/or metastatic spread after surgery have been seen in 40–90% of all cases treated surgically [12].

Over 95 percent of GIST cells have mutations in one of the two genes, called *KIT (CD117)* and *PDGFR*α** [12, 13]. The drug imatinib targets both of these mutated genes and block cellular communications that result in tumor growth. Clinical trials have shown that imatinib can kill GIST cells that have spread (metastasized) to other parts of the body and cannot be removed with surgery. Tumor cells depend on activation of aberrant growth signal pathways for both proliferation and survival [13]. These two phenomena are intricately tied together rather than existing as separate events [14]. In many experimental models the withdrawal of a growth factor pathway stimulatory signal leads to cell death by a mechanism such as apoptosis. The treatment of a “Kit-driven” GIST cell with imatinib effectively results in withdrawal of growth factor support. Thus, there is rationale behind the idea that inhibition of Kit signaling may inactivate molecules that are needed for GIST cell survival [13, 14].

Imatinib mesylate was first approved by the FDA in 2001. Imatinib mesylate is the first and only effective drug for the treatment of gastrointestinal stromal tumor at present. Mutated exon 11 of the KIT receptor is essential for the pathogenesis and response to imatinib mesylate of gastrointestinal stromal tumor. The efficacy rate (complete response + partial response) of imatinib mesylate is 53.8%, and the disease-control rate (complete response + partial response + stable disease) is 84% [15]. Clinical trials suggest that an increased dose of imatinib mesylate would be beneficial, and that the interruption of imatinib treatment might result in disease progression even after a partial response. The latest results for imatinib in GIST come from the BRF14 trial conducted by the French Sarcoma Group. In this trial, after a median followup of 35 months, patients who had stopped therapy had a significantly higher risk for rapid progression than those who continued taking the drug. The 2-year progression-free survival was 80% in those who continued taking imatinib, compared with 16% in those who stopped (*P* < .0001). Treatment with imatinib should not be stopped in patients with advanced GIST, because interruption of therapy places them at a high risk for rapid progression of the disease [15].

As per National Cancer Institute recommendations for the GIST, patients with tumor size larger than 10 centimeters, which are labelled as histopathologically high-risk category, benefited more from imatinib than those with smaller tumors. As per ASCO-2010, and the trial by Nilsson et al. indicates that 1 year of adjuvant treatment with imatinib 400 mg/day dramatically improves recurrence-free survival [4]. Imatinib as such is recommended in metastatic, residual, or recurrent cases of GISTs or which are surgically not removable; however, recent recommendations suggests the use of imatinib mesylate after radical surgery in high-risk cases, because it has shown 14% absolute decrease in the recurrence rate (97% of the patients receiving imatinib were free of recurrence (PFS) compared to 83% in the placebo group) [16]. In December 2008, the Food and Drug Administration (FDA) approved the use of imatinib as adjuvant therapy for adult patients after complete resection of localized, primary GIST.

## 4. Conclusion

Giant GISTs of the jejunum are rare tumors of the digestive tract. In cases of jejunal GIST, surgery should be considered as primary treatment. Better understanding of the cell of origin and immunohistochemical markers has made timely targeted therapy possible in GIST. Their treatment has been revolutionized with the advent of targeted molecular therapy, namely, imatinib mesylate. The therapy of advanced GIST with imatinib mesylate has been markedly successful whether by cytostatic, cytotoxic, or both mechanisms. As per latest ASCO guidelines, recurrence-free survival is increased in patients who take one year of imatinib 400 mg/day [4]. Imatinib as such is recommended in metastatic, residual, or recurrent cases of GISTs or which are surgically not removable; however, recent recommendations suggests the use of imatinib mesylate as adjuvant therapy after radical surgery in high-risk cases, because it has shown significant decrease in the recurrence rate [16]. Treatment with imatinib should not be stopped in patients with advanced GIST, because interruption of therapy places them at a high risk of rapid progression of the disease [15, 16] and patients with tumors larger than 10 cms benefited more from imatinib than those with smaller tumors.
